# Equal tibial component fixation of a mobile-bearing and fixed-bearing medial unicompartmental knee arthroplasty: a randomized controlled RSA study with 2-year follow-up

**DOI:** 10.1080/17453674.2019.1639965

**Published:** 2019-07-11

**Authors:** Daan Koppens, Søren Rytter, Stig Munk, Jesper Dalsgaard, Ole G Sørensen, Torben B Hansen, Maiken Stilling

**Affiliations:** a Department of Orthopedic Surgery, University Clinic for Hand, Hip and Knee Surgery, Hospital Unit West Holstebro, Denmark;; b Department of Orthopedic Surgery, Aarhus University Hospital, Aarhus, Denmark

## Abstract

Background and purpose — Differences in stress distribution in a mobile-bearing and fixed-bearing unicompartmental knee arthroplasty (UKA) design might lead to a difference in fixation of the tibial component. We compared tibial component migration of a mobile-bearing (MB) UKA and a fixed-bearing (FB) UKA using radiostereometric analysis.

Patients and methods — In a randomized, patient-blinded clinical trial 62 patients received either the MB Oxford UKA or the FB Sigma UKA. The patients were followed for 24 months with radiostereometric analysis. Clinical outcome was assessed with Oxford Knee Score (OKS), RAND-36 and leg extension power.

Results — Migration of the tibial components was similar between groups throughout follow-up. At 12 months, MTPM of the tibial component was 0.44 mm (95% CI 0.34–0.55) for the MB group and 0.40 mm (CI 0.31–0.50) for the FB group. Between 12 and 24 months, the tibial components migrated with a median MTPM increase of 0.03 mm (CI –0.02 to 0.08) in the MB group and 0.03 mm (CI –0.02 to 0.07) in the FB group. Continuous migration of the tibial component was found for 1 MB UKA and 2 FB UKAs. Both groups showed similar and clinically relevant improvement in clinical outcome.

Interpretation — MB and FB tibial components had similar good fixation and clinical improvement until 2 years. Based on this study, a low 5- to 10-year revision rate can be expected for both implants.

Unicompartmental knee arthroplasty (UKA) has shown good clinical outcome and implant survival for patients with medial osteoarthritis (OA) (Cheng et al. 2013, Peersman et al. 2015). The mobile-bearing (MB) medial Oxford UKA (Zimmer Biomet, Bridgend, UK) is a well-documented UKA and offers good functional results (Pandit et al. 2011, 2015), and a low 10-year revision rate of 7% and 15-year revision rate of 11% (Mohammad et al. 2018). The fixed-bearing (FB) medial Sigma UKA (DePuy International, Leeds, UK) offers 5-year revision rates between 4.7% and 5.6% in national arthroplasty registries (AOANJRR 2018, NJR 2018). Long-term results of the Sigma UKA are unknown. In 30–40% of UKA revisions, the reason is aseptic loosening (AOANJRR 2018, SKAR 2018).

A fully congruous bearing design of the MB UKA results in low contact stress. The stress of the femur on the tibia occurring during movement is transformed into an evenly distributed compressive stress at the tibial implant/bone interface. Possible disadvantages of an MB design are backside wear and dislocation of the bearing. The concave bearing design of the FB UKA results in higher contact stress, resulting in shear stress and unevenly distributed compressive stress at the bone/implant interface during loaded knee motion (Goodfellow et al. 2015). These differences in design and stress loading on the tibial bone could affect tibial component fixation.

Implant fixation can be evaluated as component migration by radiostereometric analysis (RSA), which is a predictor for late implant loosening (Ryd et al. 1995, Pijls et al. 2012b, 2018). Low early implant migration has been related to low 5- and 10-year revision rates in national registries (Pijls 2012b, 2018).

Besides implant survival, patient satisfaction and knee function are important clinical outcomes after knee surgery. Implants introduced to the market should offer at least the same clinical outcome as established implants.

We compared the MB Oxford UKA and the FB Sigma UKA with tibial component migration as the primary outcome and clinical outcome scores as a secondary outcome. We hypothesized that there was no difference in migration or clinical outcome between implants. 

## Patients and methods

**Figure 1.**
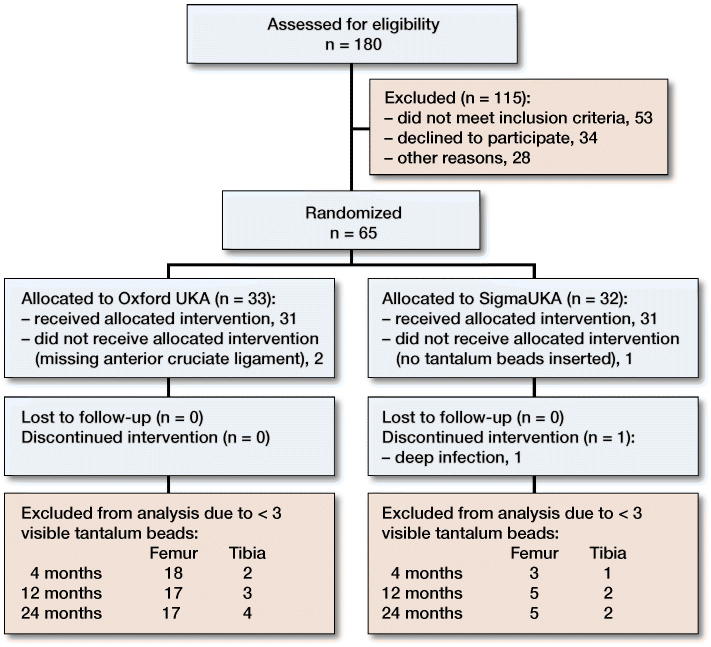
Consort 2010 flow diagram.

Between January 2014 and November 2015, a randomized, patient-blinded clinical trial was performed. Patients with primary medial OA of the knee were assessed for eligibility (Figure 1).

The inclusion criteria were patients above 18 years of age, who were eligible for medial UKA (Murray et al. 1998, DePuy International 2009). The exclusion criteria were inflammatory arthritis, contralateral knee prosthesis, disseminated malignant disease, serious systemic disease, female patients of reproductive age, and patients unable to give written informed consent.

Patients were randomized to receive the Oxford UKA (MB group) or the Sigma UKA (FB group). Randomization was done in blocks of 10 patients, generated via www.random.org/lists. Opaque envelopes were drawn 1 day before surgery for logistic reasons. If a patient was excluded during the inclusion period of the study, an extra patient was included to maintain the power of the study.

The study is reported in accordance with the CONSORT guidelines as well as the guidelines for standardization of RSA (Valstar et al. 2005) and the ISO standard for RSA (ISO 2013).

### Surgery and implants

The MB phase 3 Oxford medial UKA consists of a 2-pegged femoral component with a spherical articulation, a fully congruous mobile bearing, and a tibial component with a flat articulation surface and a keel at the non-articulating surface. The FB Sigma medial UKA consists of a 2-pegged femoral component with a large posterior condyle radius, a concave fixed bearing, and a tibial component with a keel and a peg at the non-articulating surface. Both UKAs were implanted with Palacos bone cement (Heraeus Holding GmbH, Hanau, Germany). 2 orthopedic surgeons (SM and JD) experienced with FB and MB UKA performed the surgeries. The manufacturer’s instructions were followed, and a minimally invasive approach was used. During surgery, 4 to 6 1-mm tantalum beads were inserted in the periprosthetic femoral and tibial bone in order to accommodate future RSA analysis. All patients followed a fast-track program (Koppens et al. 2018).

### Primary outcome

#### Radiostereometric analysis

A previously described standardized RSA set-up (Koppens et al. 2018) with the patient supine was used to obtain stereoradiographs on the first postoperative day, and at 4, 12, and 24 months. An auto-positioning, direct-digital roentgen system (AdoraRSA suite, NRT, Aarhus, Denmark) was used. 2 ceiling-fixed, synchronized roentgen tubes (Varian Medical Systems, Palo Alto, CA, USA) were positioned 100 cm above the calibration box at an angle of 40° to each other. Digital image detectors (Canon, CXDi-701C Wireless; Canon Europe, Uxbridge, UK) were placed behind the calibration box. Digital radiographs were stored in DICOM format at a resolution of 160 µm pixel pitch and a 16-bit grey-scale resolution in a picture archiving and communication system (PACS).

All analyses were performed with Model-Based RSA software version 4.11 by use of computer-aided design (CAD) models (RSAcore, Leiden, The Netherlands). The upper limit of mean error rigid body fitting was 0.35 mm, and 120 for the condition number (Valstar et al. 2005, ISO 2013). If migration analysis was not possible due to occluded markers or primary analysis showed a high condition number (> 80), a patient-specific marker configuration model (MC model) of the bone markers was constructed if possible and applied in the analysis (Kaptein et al. 2005). An MC model for the tibial bone was used to analyze 3 tibial components in the MB group and 4 tibial components in the FB group. An MC model for the femoral bone was used to analyze 6 femoral components in the MB group and 7 femoral components in the FB group. Patients with less than 3 visible markers were excluded. The postoperative stereoradiograph served as reference examination. The y-axis of the calibration box was parallel to the anatomical axis of the leg. Signed translations along and rotations around the x-, y-, and z-axis were defined as Tx (lateral/medial), Ty (distal/proximal), and Tz (posterior/anterior) and as Rx (flexion/extension), Ry (external/internal), and Rz (abduction/adduction) (Valstar et al. 2005).

Total translation (TT) for the center of gravity of the implant was defined as: √(*Tx^2^ × Ty^2^ × Tz^2^*)

For small rotations, total rotation (TR) can be defined as (Kaptein et al. 2007): √(*Rx^2^ × Ry^2^ × Rz^2^*)

Maximal total point motion (MTPM) was defined as the translation vector of the point in the CAD model that had the greatest motion (Valstar et al. 2005). Continuous migration was defined as MTPM more than 0.2 mm between 12 and 24 months (Ryd et al. 1995).

#### Precision of RSA

The precision of the measurements was based on double examinations on all patients taken at 12 months’ follow-up. The postoperative stereoradiograph was used as a reference in the migration analysis. The bias was defined as the mean difference in translation along and the rotation about the three axes between the double examinations. The precision was defined as the standard deviation (SD) of the difference (SD_dif_). The expected clinical precision was represented as the prediction interval (PI) and defined as 1.96 x SD_dif_.

Pooled data were comparable to precision data from the literature (Tables 1 and 2, see Supplementary data) (Stilling et al. 2011, Molt et al. 2012, Pijls et al. 2012a, Koppens et al. 2018).

**Table 3.** Summary of baseline characteristics

Factor	MB UKA (n = 33)	FB UKA (n =32)
Mean age (range)	64 (50–78)	61 (47–79)
Male/female sex	16/17	17/15
Mean weight, kg (SD)	87 (15)	89 (13)
Mean height, cm (SD)	171 (10)	173 (9)
Mean BMI (SD)	29 (4)	30 (4)
Mean Oxford Knee Score (SD)	26 (4.8)	28 (7.1)
RAND-36 (SD)		
physical functioning	50 (17)	53 (19)
pain	65 (44)	72 (38)
general health	74 (18)	75 (14)

**Table 8.** Oxford Knee Score (mean (CI)), ranging from 0 (worst) to 48 (best)

Time	MB UKA	FB UKA
Preoperative	26 (24–28)	28 (26–30)
4 months	38 (35–40)	37 (34–39)
12 months	42 (40–44)	41 (39–43)
24 months	40 (37–43)	41 (38–44)

### Secondary outcome

#### PROMs

The Oxford Knee Score (OKS) (Murray et al. 2007) and a general health questionnaire (RAND-36) (Hays and Morales 2001) were obtained before surgery, and at 4, 12, and 24 months after surgery. OKS is a 12-item questionnaire, with scores ranging from 0 (worst) to 48 (best) (Odgaard and Paulsen 2009). RAND-36 was scored using the RAND scoring rules, ranging from 0 (lowest) to 100 (highest) (Laucis et al. 2015). Summary scores for physical functioning, role limitations caused by physical health problems, pain, and general health perception were given.

#### Leg-extension power

Functional outcome was measured as the leg-extensor power (LEP) (Bassey and Short 1990) using the leg-extensor power rig (Bio-Med International, Nottingham, UK) (Barker et al. 2012, Munk et al. 2012). Both legs were tested before surgery and at 24 months after surgery, and the operated leg was further tested at 1 and 12 months after surgery. Patients performed a minimum of 5 repetitions and a maximum of 10 repetitions. The session was stopped if the patient had reached his or her maximum, defined as 2 attempts with a lower score than the previous or if the patient reported pain in the knee (Munk et al. 2012). The maximum recorded measurement was used in the analysis. LEP is expressed as power per kg of body weight (W/kg).  

### Statistics

#### Sample size

A generally accepted threshold for migration is the difference in MTPM between 12 and 24 months > 0.2 mm (Ryd et al. 1995). To detect a 0.2 mm difference in MTPM we needed 22 patients in each group (power 90%, alpha 0.05, SD 0.2 mm) (Kendrick et al. 2015). To anticipate dropouts, 30 patients were included in each group.

#### RSA

All RSA data were assessed using mixed-model analysis (MMA) (Ranstam 2012). Assumptions concerning the data distribution were ensured, using mixed-model residual QQ-plots, fitted vs. residuals plots and histograms. A likelihood-ratio test was used to detect differences between models, a Wald test to detect differences within the model. If a difference within the model was found, pairwise comparisons were used to specify the differences.

Translations and rotations were shown as mean and 95% confidence intervals (CI).

MTPM, TT, and TR were not normally distributed and were therefore analyzed on a logarithmic scale (median and CI reported). To accommodate comparison in the literature, mean and CI was also presented (MMA without logarithmic transformation).

#### PROMs

OKS was analyzed using mixed-model analysis (Ranstam 2012). The minimal clinically important difference (MCID) was defined as 9 points within groups, and 5 points between groups (Beard et al. 2015).

#### Leg-extension power

LEP data of the operated leg were analyzed using mixed model analysis. LEP data of the operated leg and the contralateral leg preoperatively and at 24 months were analyzed using paired t-tests (Barker et al. 2012). All data gathered on excluded patients were included in the analysis up to the time of exclusion (Figure 1).

Statistical significance was assumed at p < 0.05. Intercooled Stata version 13.1 (StataCorp, College Station, TX, USA) was used for statistical analysis.  

### Ethics, registration, funding, and potential conflicts of interest

The study was approved by the Central Denmark Region Committee on Biomedical Research Ethics (journal no. 1-10-72-591-12; issue date 12-03-2013) and the Danish Data Protection Agency (journal no. 1-16-02-82-13; issue date 22-05-2013). The study was conducted in accordance with the Helsinki Declaration and registered with ClinicalTrials.gov (NCT03434600). CAD implant models were provided from the implant companies. DK received a public grant for VIP salary from the Health Research Fund of Central Denmark Region. The other authors had no conflict of interest.

## Results

Baseline patient characteristics are given in Table 3.

1 patient with a FB UKA was excluded 5 weeks after primary surgery due to deep infection.

### Primary outcome—RSA

#### Tibial component

Migration of the tibial components was similar between groups throughout follow-up, although both groups showed some migration over time (Tables 4 and 5, see Supplementary data, Figure 2). Between 4 and 24 months, the tibial components showed lift-off of mean 0.05 mm (CI 0.02–0.08) in the MB group and mean 0.04 mm (CI 0.01–0.07) in the FB group. Between 4 and 12 months, the tibial components showed posterior rotation of mean –0.18° (CI –0.29 to –0.08) in the MB group and mean –0.21° (CI –0.31 to –0.11) in the FB group. Between 12 and 24 months, the tibial components migrated with a median MTPM increase of 0.03 mm (CI –0.02 to 0.08) in the MB group and 0.03 mm (CI –0.02 to 0.07) in the FB group. Continuous migration was found for 1 MB UKA and 2 FB UKAs.

**Figure 2.**
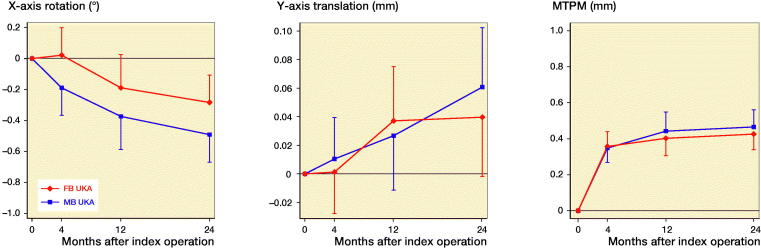
(a) X-rotation, (b) Y-translation, and (c) maximal total point motion (MTPM) for the tibial component (median and 95% CI).

#### Femoral component

Translations and rotations of the femoral components were similar between groups throughout follow-up (Table 6, see Supplementary data). At 4 months, the FB group showed a median 0.46° (CI 0.20–0.63) higher TR than the MB group. Also, the FB group showed a median 0.20 mm (CI 0.04–0.30) higher MTPM than the MB group at 4 months. This difference in TR and MTPM for the femoral component remained throughout follow-up (Table 7, Figure 3, see Supplementary data).

### Secondary outcome

#### OKS and RAND-36

OKS is shown in Table 8 and RAND-36 in Table 9 (see Supplementary data).

#### LEP

LEP was similar between groups throughout follow-up (Table 10, see Supplementary data). Preoperatively, the knee awaiting surgery had lower LEP than the contralateral knee. At 24 months, both limbs performed equally.  

## Discussion

### RSA

The MB and FB group showed similar tibial component migration with migration primarily in the first 12 months after surgery, after which the tibial components stabilized. Early migration of tibial components can be expected in the first 12 months (Kendrick et al. 2015, Koppens et al. 2018), but stabilization between 12 and 24 months is important (Ryd et al. 1995).

We thought a higher strain at the bone interface of the FB compared with the MB tibial components to be a potential risk of higher migration. However, this was not the case, and a difference in the design of the backside of the tibial components might explain this. The keel on the FB tibial component is wider than on the MB tibial component, and further there is an extra peg on the medial side of the FB tibial component, which provides extra stability.

In both the MB and FB group, the tibial component showed lift-off from the tibial bone (translation on the y-axis) until 12 months, and thereafter stabilized. This can be explained by the posterior rotation (rotation around the x-axis) of tibial components in both groups seen between 4 and 12 months. However, the posterior rotation was less than 0.8° at 24 months, which has recently been suggested as an acceptable threshold. Tibial lift-off, subsidence, and especially posterior rotation were shown to be predictors for late loosening (Gudnason et al. 2017). Signed migration measures have the advantage that they differentiate in the direction of measured migration, whereas the MTPM gives an implant- and time-dependent summarized vector-based migration measure. Although interesting, these thresholds (Gudnason et al. 2017) were based on a historical cohort of 116 patients of which only 5 were failures, and 4 of these failures had the same implant design. Combined prospective RSA data from several centers with long-term follow-up of failures are needed to validate new thresholds to be used as predictors for loosening.

Recently Pijls et al. (2018) re-defined migration thresholds based on MTPM measures, with acceptance thresholds of 0.50 mm MTPM at 6 months’ follow-up (early migration), 0.20 mm continuous MTPM between 6 and 12 months (stabilization phase I), and 0.20 mm continuous MTPM between 12 and 24 months (stabilization phase II). Both the MB and FB tibial components showed acceptable migration on group level during stabilization phases I and II. Continuous migration was shown only for 1 MB and 2 FB tibial components. These results are in line with the low registry-reported short- to mid-term revision rates of both components, which is 6–8.4% for MB UKA and 4.7–5.6% for FB UKA at 5 years’ follow-up (AOANJRR 2018, NJR 2018).

Other RSA studies evaluating fixation of UKA have found tibial component migration comparable to our study. Kendrick et al. (2015) compared cemented and cementless Oxford UKA until 24 months’ follow-up and found equally low migration for both groups. For the cemented tibial component, some posterior rotation was seen between 3 and 6 months though not statistically significant. In a prospective cohort RSA study (Koppens et al. 2018), we have formerly reported low migration of the FB Sigma UKA until 2 years’ follow-up, and MTPM under the 1-year threshold defined by Pijls et al. (2012b). In this study, we also found some posterior rotation throughout the follow-up period, though below the 0.8° threshold (Gudnason et al. 2017).

The femoral component of the FB group showed a slightly higher MTPM than the MB group at 4 months, after which both groups stabilized. No thresholds exist for migration of the femoral component; migration of the femoral component in the FB group was similar as previously reported (Koppens et al. 2018).

### Clinical outcome

The MB and FB UKA had an equally good clinical outcome. Both groups experienced a statistically significant and clinically relevant (Beard et al. 2015) improvement in knee pain and function from poor preoperatively to good up to 12 months after surgery. This improvement was sustained up to 24 months after surgery. Comparable improvements have been shown in the literature for both MB UKA (Pandit et al. 2011, Kendrick et al. 2015) and FB UKA (Koppens et al. 2018). Overall, the general health improved equally in both groups after surgery. Clinically relevant improvements were shown after surgery for “physical function,” “role limitations due to physical health,” and “pain” (Keurentjes et al. 2014).

An improvement in LEP over time was observed in both groups, and after 24 months there was no inter-limb difference (operated vs. non-operated leg) in LEP. Similar improvements in LEP have been seen in patients with UKA (Barker et al. 2012, Jorgensen et al. 2017).

### Limitations

Some limitations should be noted. First, nearly 20% of eligible patients declined to participate in the study, which could have resulted in selection bias. However, this decline rate is not unusual for surgical trials (Thoma et al. 2010). Our results should therefore be generalizable to other similar clinics. Second, a number of the stereoradiographs were unsuitable for analysis due to occluded markers. This issue was partly solved by using an MC model (Kaptein et al. 2005). Third, non-weightbearing stereoradiographs were taken, which might have given an underestimation of tibial subsidence (Horsager et al. 2017). This is, though, not of influence on the comparison made in this study.

In summary, the MB and FB tibial components had similar good fixation and clinical improvement until 2 years, and therefore a low long-term (5–10 year) revision rate can be expected for both implants.

### Supplementary data

Tables 1, 2, 4–7, 9, and 10 as well as Figure 3 are available as supplementary data in the online version of this article, http://dx.doi.org/10.1080/17453674.2019.1639965

## Supplementary Material

Supplemental Material
