# Supplementary material for: Equal tibial component fixation of a mobile-bearing and fixed-bearing medial unicompartmental knee arthroplasty: a randomized controlled RSA study with 2-year follow-up
Source: Acta Orthop. 2019 Jul 11;90(6):575–81. doi: 10.1080/17453674.2019.1639965 (PMC6844442; doi:10.1080/17453674.2019.1639965)
Supplement: Supplemental Material [file IORT_A_1639965_SM0702.pdf]

Supplementary data

Table 1. RSA measurement repeatability of the tibial component (MB UKA and FB UKA combined) (n = 55)

Factor	Translations (mm) x	y	z	Rotations (°) x	y	z
Mean difference	0.01	0.00	0.01	0.00	0.03	0.03
SD of difference	0.05	0.03	0.09	0.18	0.16	0.14
PI (1.96 x SD)	0.1	0.07	0.18	0.36	0.32	0.27
Minimum difference	–0.19	–0.14	–0.21	–0.38	–0.72	–0.42
Maximum difference	0.22	0.05	0.37	0.71	0.31	0.44

Table 2. RSA measurement repeatability of the femoral component (MB UKA and FB UKA combined) (n = 37)

Factor	Translations (mm) x	y	z	Rotations (°) x	y	z
Mean difference	0.03	0.00	0.02	0.00	0.05	0.12
SD of difference	0.11	0.05	0.19	0.29	0.28	0.39
PI (1.96 x SD)	0.21	0.09	0.37	0.57	0.54	0.77
Minimum difference	–0.25	–0.07	–0.78	–0.61	–0.51	–1.03
Maximum difference	0.30	0.18	0.27	0.91	0.89	0.80

Table 4. Translations along and rotations about the x-, y-, and z-axis for the tibial component (mean and CI)

	MB UKA	FB UKA
Tx (mm)		
4 months	0.06 (0.02 to 0.11)	0.03 (–0.01 to 0.08)
12 months	0.09 (0.03 to 0.15)	0.04 (–0.02 to 0.10)
24 months	0.08 (0.03 to 0.13)	0.05 (–0.00 to 0.10)
Ty (mm)		
4 months	0.01 (–0.02 to 0.04)	0.00 (–0.03 to 0.03)
12 months	0.03 (–0.01 to 0.06)	0.04 (–0.00 to 0.08)
24 months	0.06 (0.02 to 0.10)	0.04 (–0.00 to 0.08)
Tz (mm)		
4 months	–0.08 (–0.16 to –0.01)	0.00 (–0.07 to 0.08)
12 months	–0.11 (–0.20 to –0.01)	0.03 (–0.07 to 0.12)
24 months	–0.08 (–0.16 to –0.01)	0.03 (–0.04 to 0.11)
Rx (°)		
4 months	–0.19 (–0.36 to –0.01)	0.02 (–0.15 to 0.20)
12 months	–0.37 (–0.59 to –0.16)	–0.19 (–0.40 to 0.03)
24 months	–0.49 (–0.67 to –0.31)	–0.28 (–0.46 to –0.11)
Ry (°)		
4 months	0.04 (–0.17 to 0.24)	–0.17 (–0.38 to 0.04)
12 months	0.02 (–0.19 to 0.24)	–0.28 (–0.50 to –0.07)
24 months	0.02 (–0.20 to 0.24)	–0.25 (–0.47 to –0.03)
Rz (°)		
4 months	–0.10 (–0.22 to 0.02)	–0.03 (–015 to 0.09)
12 months	–0.18 (–0.36 to –0.00)	0.06 (–0.12 to 0.25)
24 months	–0.18 (–0.38 to 0.01)	0.01 (–0.18 to 0.21)

Table 6. Translations along and rotations about the x-, y-, and z-axis for the femoral component (mean and CI)

	MB UKA	FB UKA
Tx (mm)		
4 months	0.02 (–0.07 to 0.12)	–0.05 (–0.12 to 0.02)
12 months	0.05 (–0.06 to 0.15)	–0.05 (–0.13 to 0.02)
24 months	–0.02 (–0.12 to 0.07)	–0.06 (–0.13 to 0.00)
Ty (mm)		
4 months	0.03 (–0.04 to 0.09)	0.07 (0.02 to 0.11)
12 months	0.02 (–0.04 to 0.09)	0.06 (0.01 to 0.11)
24 months	0.01 (–0.07 to 0.09)	0.07 (0.02 to 0.13)
Tz (mm)		
4 months	0.12 (–0.00 to 0.25)	0.05 (–0.04 to 0.14)
12 months	0.15 (0.02 to 0.27)	0.02 (–0.07 to 0.11)
24 months	0.15 (0.02 to 0.27)	0.01 (–0.08 to 0.11)
Rx (°)		
4 months	–0.04 (–0.37 to 0.28)	0.21 (–0.02 to 0.45)
12 months	0.08 (–0.23 to 0.40)	0.27 (0.04 to 0.50)
24 months	0.17 (–0.13 to 0.47)	0.40 (0.18 to 0.62)
Ry (°)		
4 months	0.19 (–0.12 to 0.49)	0.38 (0.16 to 0.60)
12 months	0.28 (–0.03 to 0.59)	0.42 (0.19 to 0.64)
24 months	0.38 (0.07 to 0.69)	0.53 (0.30 to 0.75)
Rz (°)		
4 months	–0.11 (–0.43 to 0.21)	–0.26 (–0.49 to –0.03)
12 months	–0.06 (–0.41 to 0.29)	–0.10 (–0.36 to 0.16)
24 months	–0.22 (–0.56 to 0.12)	–0.14 (–0.39 to 0.11)

Table 5. TT, TR and MTPM for the tibial component, expressed as median (CI) and mean (CI)

	Median (CI) MB UKA	FB UKA	Mean (CI) MB UKA	FB UKA
TT (mm)				
4 months	0.16 (0.12–0.20)	0.18 (0.13–0.22)	0.21 (0.15–0.26)	0.22 (0.16–0.28)
12 months	0.20 (0.14–0.25)	0.18 (0.13–0.23)	0.26 (0.17–0.35)	0.25 (0.17–0.34)
24 months	0.20 (0.15–0.24)	0.19 (0.14–0.23)	0.25 (0.18–0.31)	0.23 (0.17–0.29)
TR (°)				
4 months	0.51 (0.37–0.64)	0.48 (0.35–0.60)	0.61 (0.41–0.81)	0.66 (0.46–0.86)
12 months	0.59 (0.43–0.75)	0.60 (0.43–0.76)	0.76 (0.52–1.01)	0.80 (0.55–1.05)
24 months	0.69 (0.54–0.85)	0.65 (0.51–0.79)	0.81 (0.58–1.04)	0.83 (0.60–1.06)
MTPM (mm)				
4 months	0.35 (0.27–0.43)	0.36 (0.28–0.44)	0.42 (0.31–0.53)	0.44 (0.33–0.55)
12 months	0.44 (0.34–0.55)	0.40 (0.31–0.50)	0.54 (0.40–0.69)	0.51 (0.37–0.66)
24 months	0.47 (0.37–0.56)	0.43 (0.34–0.51)	0.55 (0.43–0.67)	0.50 (0.38–0.62)

Table 7. TT, TR and MTPM for the femoral component, expressed as median (CI) and mean (CI)

	Median (CI)		Mean (CI)	
	MB UKA	FB UKA	MB UKA	FB UKA
TT (mm)				
4 months	0.21 (0.14–0.29)	0.25 (0.18–0.29)	0.21 (0.15–0.26)	0.22 (0.16–0.28)
12 months	0.23 (0.14–0.31)	0.24 (0.17–0.31)	0.26 (0.18–0.35)	0.25 (0.17–0.35)
24 months	0.22 (0.14–0.29)	0.27 (0.20–0.33)	0.25 (0.18–0.31)	0.23 (0.17–0.29)
TR (°)				
4 months	0.50 (0.33–0.67)	0.96 (0.73–1.20)	0.61 (0.41–0.81)	0.66 (0.46–0.86)
12 months	0.65 (0.41–0.89)	0.90 (0.65–1.14)	0.76 (0.52–1.01)	0.80 (0.55–1.05)
24 months	0.58 (0.36–0.81)	0.94 (0.67–1.20)	0.81 (0.58–1.04)	0.83 (0.60–1.06)
MTPM (mm):				
4 months	0.38 (0.28–0.49)	0.58 (0.47–0.68)	0.42 (0.31–0.53)	0.44 (0.33–0.55)
12 months	0.45 (0.32–0.59)	0.55 (0.43–0.67)	0.54 (0.40–0.69)	0.51 (0.37–0.66)
24 months	0.42 (0.30–0.53)	0.61 (0.49–0.74)	0.55 (0.43–0.67)	0.50 (0.38–0.62)

Table 9. RAND-36 summary scores (mean (SD)) over time

	MB UKA	FB UKA
Physical function		
Preoperative	50 (17)	53 (19)
4 months	74 (17)	75 (14)
12 months	78 (16)	82 (11)
24 months	78 (19)	81 (14)
Limitations/physical health		
Preoperative	37 (39)	43 (36)
4 months	57 (43)	67 (40)
12 months	74 (39)	74 (40)
24 months	74 (40)	84 (29)
Pain		
Preoperative	65 (44)	72 (38)
4 months	77 (38)	87 (32)
12 months	85 (36)	81 (34)
24 months	87 (32)	91 (23)
General health		
Preoperative	74 (18)	75 (14)
4 months	78 (17)	80 (14)
12 months	73 (18)	81 (18)
24 months	76 (18)	81 (19)

Table 10. Leg extension power (mean and CI) for the operated leg and the contralateral leg

	MB UKA	FB UKA
Operated leg (W/kg)		
Preoperative	1.5 (1.3–1.7)	1.7 (1.5–1.8)
4 months	1.4 (1.2–1.6)	1.5 (1.3–1.7)
12 months	2.0 (1.8–2.2)	1.9 (1.6–2.1)
24 months	1.8 (1.6–2.0)	1.9 (1.7–2.1)
Non-operated leg (W/kg)		
Preoperative	1.9 (1.7–2.2)	2.1 (1.8–2.4)
24 months	1.8 (1.6–2.0)	1.9 (1.6–2.1)

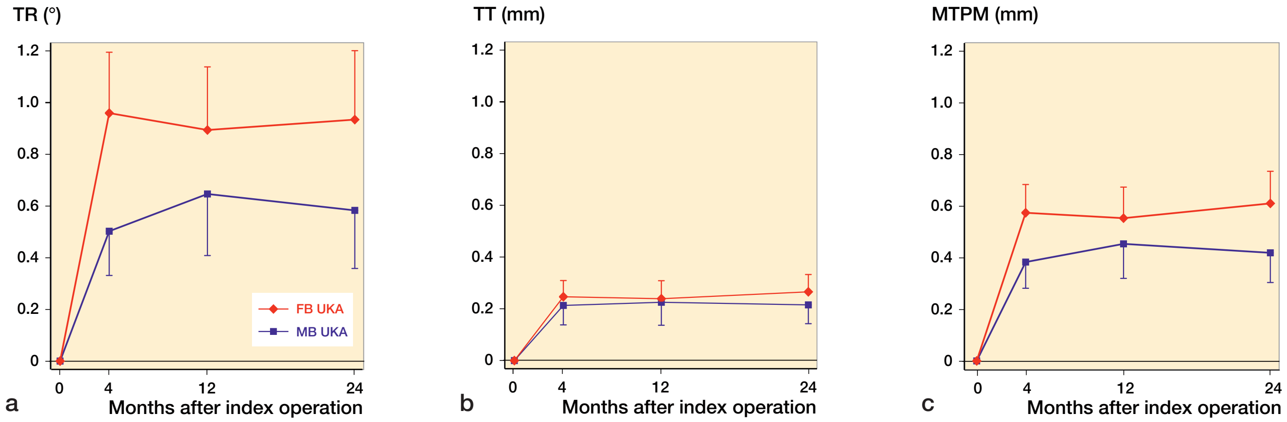

Figure 3. (a) total rotation (TR), (b) total translation (TT), and (c) maximal total point motion (MTPM) for the femoral component (median and CI).
